# End-to-End Multi-Scale Adaptive Remote Sensing Image Dehazing Network

**DOI:** 10.3390/s25010218

**Published:** 2025-01-02

**Authors:** Xinhua Wang, Botao Yuan, Haoran Dong, Qiankun Hao, Zhuang Li

**Affiliations:** 1 School of Computer Science, Northeast Electric Power University, Jilin 132012, China; wangxh@neepu.edu.cn (X.W.); 2202100980@neepu.edu.cn (B.Y.); 2022307020114@neepu.edu.cn (H.D.); 2202100963@neepu.edu.cn (Q.H.); 2 State Key Laboratory of Applied Optics, Changchun Institute of Optics, Fine Mechanics and Physics, Chinese Academy of Sciences, Changchun 130033, China

**Keywords:** remote sensing for defogging, dilated convolution, self-adaptive attention, multi-scale feature extraction

## Abstract

Satellites frequently encounter atmospheric haze during imaging, leading to the loss of detailed information in remote sensing images and significantly compromising image quality. This detailed information is crucial for applications such as Earth observation and environmental monitoring. In response to the above issues, this paper proposes an end-to-end multi-scale adaptive feature extraction method for remote sensing image dehazing (MSD-Net). In our network model, we introduce a dilated convolution adaptive module to extract global and local detail features of remote sensing images. The design of this module can extract important image features at different scales. By expanding convolution, the receptive field is expanded to capture broader contextual information, thereby obtaining a more global feature representation. At the same time, a self-adaptive attention mechanism is also used, allowing the module to automatically adjust the size of its receptive field based on image content. In this way, important features suitable for different scales can be flexibly extracted to better adapt to the changes in details in remote sensing images. To fully utilize the features at different scales, we also adopted feature fusion technology. By fusing features from different scales and integrating information from different scales, more accurate and rich feature representations can be obtained. This process aids in retrieving lost detailed information from remote sensing images, thereby enhancing the overall image quality. A large number of experiments were conducted on the HRRSD and RICE datasets, and the results showed that our proposed method can better restore the original details and texture information of remote sensing images in the field of dehazing and is superior to current state-of-the-art methods.

## 1. Introduction

In the field of computer vision, remote sensing image dehazing is one of the most popular research directions. The blurring of images is caused by the presence of suspended particles such as dust and smoke in the atmosphere, which absorb and scatter light, resulting in a thin layer of haze that reduces visual quality. This can significantly affect the performance of various computer vision tasks, such as remote sensing image object detection and recognition.

At present, the mainstream image defogging research is mainly divided into the following types. One is the image defogging method combining atmospheric scattering model and deep learning [1]. Through the estimation of transmission map and atmospheric light, and then through the training of network model, the defogging image is finally obtained. However, because the large atmospheric scattering model itself is a highly simplified physical model, the atmospheric light and transmission map are predicted in deep learning; there will be large errors, and the defogging effect is not ideal. The other is to estimate the atmospheric light value and transmittance without relying on the atmospheric scattering model. It is an end-to-end image defogging method, which can directly learn the features from the image and countermeasure network (GAN), and is widely used [2]. By training a generator and a discriminator, the defogging image is outputted. At the same time, there are some defogging methods based on deep convolutional neural network (DCNN), such as residual network and densenet [3]. Many studies show that the end-to-end deep learning method achieves better defogging effect than the method based on physical model and prior knowledge.

A key aspect of remote sensing image dehazing is multi-scale feature extraction [4]. The capture of different information by features at different scales plays an important role in the comprehensive understanding of the scene. Multiple methods have been proposed to extract multi-scale features, including pyramid-based methods and multi-scale convolutional neural networks (CNN). These methods aim to capture local details and global contextual information, achieving a more comprehensive representation of remote sensing images. In this study, we utilized dilated convolutions with different dilation rates for multi-scale feature extraction [5]. Dilated convolution shows good performance in capturing multi-scale information without significantly increasing computational complexity. By using dilated convolutions with different dilation rates, features can be extracted at multiple scales, including subtle local details and a broader global context. The choice of different expansion rates allows us to control the receptive field of each convolutional layer and captures information at different scales. This flexibility enables us to adaptively extract important features at different granularity levels, effectively addressing the challenges of scale changes in remote sensing images. In addition, we also use feature fusion technology to integrate the extracted multi-scale features, promoting more comprehensive representation and enhancing the restoration of details in dehazing images.

The attention mechanism [6] can automatically adjust the weights of each pixel based on the content and contextual information of the image, thereby paying more attention to important features and regions. This can effectively improve the effect of image dehazing and reduce interference with irrelevant information. Self-adaptive attention mechanism is a commonly used attention mechanism that can automatically learn the weights of each position based on image features and contextual information. In remote sensing image dehazing, the self-adaptive attention mechanism can adaptively adjust weights based on different regions and features of the image and focus more attention on important details and structures. This can improve the ability of the dehazing model to recover details in remote sensing images and reduce the impact on noise and irrelevant information. By introducing a self-adaptive attention mechanism, our method can better capture important detailed information in remote sensing image dehazing. The self-adaptive attention mechanism can automatically adjust weights based on the characteristics and content of remote sensing images, allowing the model to pay more attention to key details and structures in the image. This can improve the defogging effect, restore important information obscured by haze in remote sensing images, and provide clearer and more accurate image results.

In this paper, we have the following three contributions:

Haze remote sensing image dataset: Preprocess the HRRSD dataset and synthesize a haze remote sensing image dataset based on atmospheric scattering models using atmospheric light values and transmittance for model training and evaluation. Set the atmospheric light value parameters of the atmospheric scattering model between 0.75 and 1.0 and the transmittance between 0.25 and 0.75 and generate three different degrees of haze remote sensing images based on different atmospheric light values and transmittance: mild, moderate, and severe.

Self-adaptive attention mechanism: Introduce a self-adaptive attention mechanism into the model, for feature maps of different scales in the channel dimension. The weight of the channel is adaptively adjusted to effectively capture important feature information of different scales and improve the expression ability of the features.

Multi-scale adaptive feature extraction module: Propose a multi-scale adaptive feature extraction module. Then, adjust the model to reach its optimal state by increasing the number of modules and changing the network depth. These feature extraction modules can capture the details and structure of images at different scales and use self-adaptive attention mechanisms to automatically adjust weights, highlight key detailed information, thereby improving the dehazing effect and obtaining clearer and more accurate image restoration results.

## 2. Related Works

The goal of remote sensing image dehazing is to improve the visibility and contrast of the image by eliminating particles such as water vapor and suspended particles in the atmosphere. In practice, people usually adopt two main methods to achieve this goal: physical model-based methods and deep learning-based methods. The method based on physical models utilizes atmospheric light transmission models to describe the propagation process of light in the atmosphere. These methods require first estimating parameters such as atmospheric light and transmittance, and then restoring the original image through deconvolution or optimization algorithms. These methods are based on the accurate modeling of atmospheric light transmission processes and can effectively remove the influence of atmospheric shading. However, methods based on physical models often require accurate prior information and complex computational processes, which may have certain limitations for complex scenarios and changing atmospheric conditions. In contrast, deep learning-based methods directly learn the mapping relationship of image dehazing by training convolutional neural networks (CNNs). These methods can automatically learn feature representations and dehaze maps in images through a large amount of annotated data and end-to-end training of deep networks. This data-driven approach has strong flexibility and generalization ability and can adapt to different remote sensing images and atmospheric conditions. In addition, deep learning-based methods typically have fast processing speed and low computational complexity, making them suitable for real-time applications and large-scale data processing.

### 2.1. Physical Model-Based Remote Sensing Image Dehazing

The early dehazing models used atmospheric scattering models to solve the problem of haze removal and established models based on the process of foggy imaging. It consists of an incident light attenuation model and an atmospheric light imaging model. The dehazing process uses prior knowledge or estimates multiple unknown parameter variables in the model, and then directly derives the fog image from the foggy image. The DehazeNet proposed by Cai et al. [7] was the first to apply convolutional neural networks to dehazing tasks. The network takes foggy images as input and outputs predicted transmittance maps, reconstructing clear dehazing images based on atmospheric scattering models. The AOD-Net proposed by Li et al. [8] does not estimate atmospheric light values and transmission matrices separately, but directly reconstructs fog-free images through an integrated lightweight CNN. Zhang et al. [1] proposed a new dense connection encoder–decoder structure, combined with multi-level pyramid pooling modules, for estimating transmission maps. This network is optimized using a newly introduced edge loss function. To enhance the integration of structural information between the estimated transmission image and the dehazing result, a joint discriminator within a generative adversarial network framework is introduced. This discriminator assesses whether the dehazing image and the estimated transmission image are authentic or synthetic.

The combination of atmospheric scattering models and deep learning methods has significantly improved the effectiveness of image dehazing. Du et al. [9] proposed a direct deep learning method that achieves image dehazing by bypassing the transmission map estimation step through recursive deep residual learning. By introducing a discriminator and a loss function adapted to foggy conditions, the perceptual quality of dehazing images is optimized. Meanwhile, halo-like artifacts are eliminated through guided filtering. Zhao et al. [10] proposed a two-stage weakly supervised dehazing framework called RefineDNet. In the first stage, visibility is restored using a dark channel prior method. Subsequently, in the second stage, adversarial learning was performed on unpaired foggy and clear images to improve the initial dehazing results in the first stage to enhance realism. In addition, in order to achieve higher quality results, an effective perception fusion strategy has been introduced to integrate different dehazing outputs. He et al. [11] proposed a dark channel prior method for removing haze from a single input image. Based on a key observation, the local areas of most outdoor fog-free images contain pixels with very low intensity in at least one color channel. Using this prior and haze imaging model, they could directly estimate the thickness of haze and restore high-quality haze-free images. Zhu [12] created a linear model from a prior model to model the scene depth of haze images and used supervised learning methods to learn the model parameters for restoring depth information. Transmittance was estimated through depth maps of haze images and scene radiance was restored through atmospheric scattering models to effectively remove haze from the images.

### 2.2. Deep Learning-Based Remote Sensing Image Dehazing

To overcome the limitations of physical models, end-to-end network models have emerged to achieve dehazing directly. Ren et al. [13] introduced a multi-scale deep neural network to learn the mapping between blurred images and their corresponding transmission maps. This network comprises a coarse-grained component for predicting the global transmission map from the entire image and a fine-grained component for local optimization, aiming to achieve single image dehazing. Researchers proposed an algorithm based on conditional generative adversarial network cGAN [14], which introduces an encoder and decoder architecture. By introducing VGG features and regularizing gradient priors, the cGAN formula is further optimized to generate dehazing images. Engin et al. [2] proposed a cycle dehaze, which trains the network by inputting clear and blurred images in a non-paired manner. This method improves cycle GAN by combining cyclic consistency and perceptual loss, improving the quality of texture information recovery and generating haze-free images. Ma et al. [15] developed a dark channel model, which optimizes the transmission map through transmission coefficients to achieve haze removal. Zhang et al. [16] proposed a three-scale encoder and a fusion module that can efficiently and directly learn fogless images, achieving image dehazing.

With the continuous development of deep learning, many scholars have proposed numerous new dehazing models. Chen et al. [17] proposed an end-to-end-gated context aggregation network to directly recover the final fog-free image. Liu et al. [18] proposed GridDehazeNet for single image dehazing, where preprocessing, backbone, and post-processing modules work together to achieve attention-based multi-scale estimation and effectively reduce artifacts. In their subsequent research, they proposed GridDehazeNet+ [19], which is an improvement over GridDehazeNet [18], which no longer relies on atmospheric scattering models and introduces grid structures and spatial channel attention blocks, enhancing multi-scale estimation and feature fusion capabilities. Ren et al. [20] calculated pixel level confidence maps based on the appearance differences between different inputs to mix the exported input information and pre-serve regions with good visibility. Xu et al. [21] proposed the concept of “virtual depth”, where the cover plays a role in haze detection in natural images, providing clues to the foreground and background. Through iterative use of defogging operators, haze is gradually removed. Mei et al. [22] employed a progressive feature fusion UNet to directly learn the highly non-linear transformation function from observed foggy images to non-foggy real images, thereby accomplishing dehazing. In the dehazing task, generative adversarial networks (GANs) have also been widely applied. Dong et al. [23], with FD-GAN, used the proposed fusion discriminator to generate dehazing images that are more natural, realistic, with less color distortion, and fewer artifacts by incorporating frequency information into additional prior information. Dong et al. [24] proposed a multi-scale-enhanced dehazing network based on the U-Net architecture, which introduces a reinforced subtraction operation enhancement strategy in the decoder of the proposed model. By enhancing the decoder, the dehazing image is gradually restored. Huang et al. [25] proposed that cGAN combines the information of RGB and SAR images to eliminate image blur. Shao et al. [26] proposed a domain adaptive paradigm that incorporates real, haze images into dehazing training by utilizing the characteristics of clear images, further improving domain adaptability and ultimately achieving dehazing. Yin et al. [27] proposed an encoder–decoder framework, with pyramid pooling operation, and also proposed a novel parallel spatial channel attention block, which was applied at the end of the encoder to guide the decoder to reconstruct clearer images.

In the field of remote sensing, there are also many dehazing models that are specifically designed for remote sensing images. Li et al. [28] proposed a two-stage dehazing neural network, FCTF-Net, which improves the dehazing effect of remote sensing images through multi-scale feature extraction and result refinement [29]. Hong et al. [30] designed a dehazing network using a knowledge distillation method. Wu et al. [31] used the idea of contrastive learning to mine information by using fuzzy images and clear images as negative samples and positive samples, respectively. Huang et al. [32] proposed DCRD-Net, which can accurately remove haze in remote sensing images and accurately restore details. Wang et al. [33] proposed a feature pyramid-based PFE that utilizes complementary features from different CNN layers to assist in clear image prediction. Huang et al. [34] introduced self-filtering blocks to eliminate redundant features, improve the representation ability of learning features, and achieve the restoration of image content. Jin et al. [35] extracted feature representations from pre-trained DINOViT modules to recover background information, introduced uncertainty feedback learning, focused on non-uniform fog areas, and iteratively improved dehazing output based on uncertainty maps using feedback networks. Guo et al. [36] proposed SCANet, which enhances foggy occluded areas through attention generation networks and scene reconstruction networks, restricts attention maps by utilizing brightness differences in images, and ultimately achieves effective dehazing.

## 3. Method

**Figure 1:**
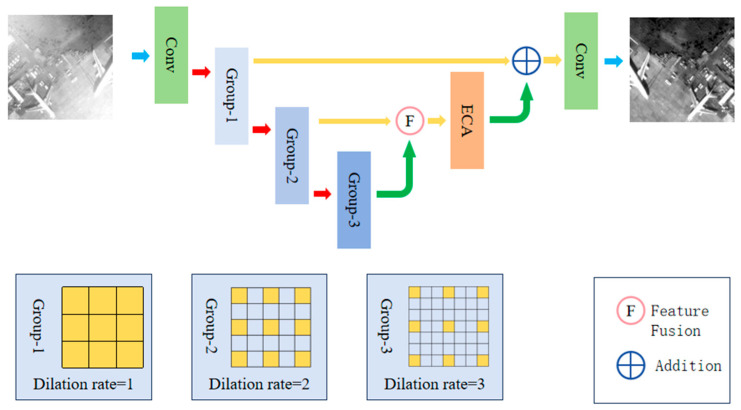
Diagram of the MSD-Net model architecture.

In this section, we will provide a detailed description of the design of the MSD-Net model. As shown in Figure 1, the model takes fog images as input. Firstly, the input image is processed through a shallow feature extraction module. Next, the image enters an architecture with multiple jump connections and multi-scale-dilated convolutional self-adaptive attention mechanisms. The multi-scale-dilated convolutional self-adaptive attention module in this architecture extracts multi-scale features from images. By performing dilated convolution operations, the model can capture the details and contextual information of images in different receptive fields. The self-adaptive attention mechanism can adaptively learn the importance of each position in the image, thereby better understanding the structure of the image.

After the feature extraction stage, we introduced a feature fusion module to fuse multi-scale feature information. The function of this module is to combine features from different scales to obtain a more comprehensive and rich feature representation. Through feature fusion, the model can better capture the details and texture information in fog images. Finally, after processing via the feature fusion module, we use the reconstruction module to restore the fog-free image. This module converts the fused feature maps into the final fog-free image. This process can be seen as an image restoration process, where the model can reduce or eliminate visual degradation caused by haze by learning the mapping from foggy images to non-foggy images.

In addition, each dilated convolutional base block structure is combined with local residual connections. This connection method allows the model to better propagate gradients during the training process and helps alleviate the problem of vanishing gradients.

**Figure 2:**
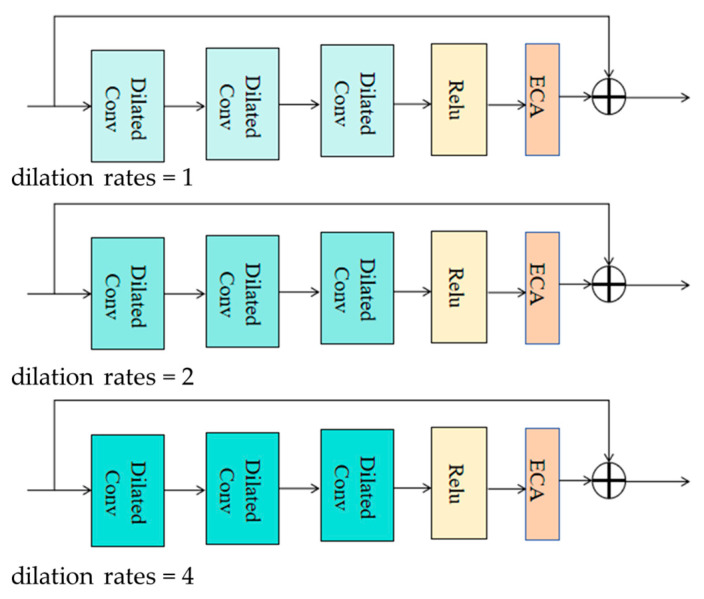
Diagram of the internal structure of the MSD-Net group module.

In Figure 2, we propose an improved group module based on feature fusion attention network to enhance its ability to obtain rich and important features. Firstly, dilated convolutions with expansion rates of 1, 2, and 4 are introduced. The three different colors at various depths represent different expansion rates, allowing for the multi-scale feature extraction of remote sensing images within different receptive field ranges, thereby enriching feature information. Secondly, the ECA attention module is introduced, which serves to adaptively extract important feature information at different scales and reduce the number of parameters in the group module. Finally, the important feature information obtained at different scales is fused to achieve a more comprehensive feature set, which enhances the clarity of the dehazing results.

### 3.1. Feature Extraction of Dilated Convolution

Multi-scale feature extraction is a commonly used technique in remote sensing image dehazing, aimed at obtaining feature information from images at different scales to better restore clear images. Among them, dilated convolutions with different expansion rates are widely used to achieve multi-scale feature extraction. Firstly, different dilation rates are introduced in the dehazing network to perform dilation convolution operations. By using dilated convolutions with dilation rates of 1, 2, and 4, feature representations of different scales are obtained. Secondly, the output feature maps of each scale’s dilated convolution will be further processed and fused. This can fuse features of different scales to obtain a more comprehensive and rich feature representation to better restore clear images.

The role of dilated convolutions with different expansion rates in remote sensing image dehazing is mainly reflected in the following aspects: (1) Multi-scale feature extraction: Dilated convolutions with different expansion rates can capture details and structural information in the image at different scales. By setting different expansion rates, receptive fields at different scales can be obtained, thereby extracting multi-scale features; it is very important to restore the details and structures of various scales in remote sensing images; (2) Enhanced feature representation: By performing dilated convolution operations with different dilation rates, feature representations from different scales can be obtained. These feature representations can capture information from different scales in the image, thereby enhancing the feature representation ability of the image, helping to more accurately restore clear images and improve the dehazing effect.

### 3.2. Self-Adaptive Attention Mechanism

Self-adaptive attention methods play an important role in remote sensing image dehazing. This method can improve the processing ability of the dehazing network for different regions in the image by introducing attention mechanisms, enabling it to more accurately perceive and reconstruct detailed information in the image. Firstly, by establishing an end-to-end dehazing model, the model receives input remote sensing images and outputs images of haze removal. Then, an attention module is introduced into the network to adaptively adjust the network’s attention level to different haze areas. The self-adaptive attention module can dynamically adjust the weights of the network at different positions based on the content and feature distribution of the image, thereby improving the perception and reconstruction ability of details. This design can help the network better handle complex scenes and differences between different regions in remote sensing images, thereby improving the dehazing effect.

The role of self-adaptive attention methods in remote sensing image dehazing is mainly reflected in the following aspects: (1) Improving detail retention ability: Self-adaptive attention methods can make the network pay more attention to the details in the image, thereby better preserving and reconstructing detailed information. By adjusting attention weights, the network can enhance its perception and reconstruction of details in a targeted manner, making the dehazing results clearer and more realistic; (2) Improving the dehazing effect: Introducing a self-adaptive attention mechanism can enable the network to more accurately perceive and process haze in images, thereby improving the dehazing effect. The attention module can automatically adjust the weight allocation of the network based on the different content of the image, enabling the network to better cope with the different degrees and types of haze. By increasing attention to haze areas, the network can better reduce the impact of haze and improve the quality and clarity of haze removal results; (3) Enhanced robustness: Self-adaptive attention methods can enhance the robustness of remote sensing image dehazing networks. By adaptively adjusting the network’s attention level, the network can better adapt to remote sensing images in different scenes and environments. This makes the network more stable and reliable in processing remote sensing images with diversity and complexity, improving processing capabilities and robustness of defogging effects.

### 3.3. Expansive Convolutional Group

In our model, the expansion basis block is composed of multiple key components, including expansion convolution, local residual learning for each expansion rate, and a self-adaptive attention module. The combination of these components enables our model to better handle remote sensing image dehazing tasks and improve model performance and training stability. 

Firstly, dilated convolution plays an important role in dilated basis blocks. By using convolution kernels with different dilation rates, dilated convolution can extract features from images within different receptive fields. This multi-scale feature extraction helps to capture details and structural information at different scales, thereby improving the dehazing effect. Meanwhile, local residual learning for each expansion rate allows the main network to bypass irrelevant information through multiple local residual connections and focus its main attention on effective information. This local residual learning strategy can enhance the network’s ability to learn key features, improving model performance and training stability. Secondly, the self-adaptive attention module plays a crucial role in expanding the base blocks. This module can automatically adjust the network’s attention level in different regions by introducing an attention mechanism. By dynamically adjusting the weight allocation of the network based on the content and feature distribution of the image, the self-adaptive attention module can improve the network’s ability to perceive and reconstruct details. This enables the network to more accurately process haze in images and improve the dehazing effect. In addition, the basic extension blocks and skip connection modules form a group, which plays an important role in MSD-Net. The ongoing addition of basic extension blocks enhances the depth and expressiveness of the network, thereby enhancing the model’s capability to model complex scenes and multi-scale information. Jumping connections make MSD-Net easier to train, which helps alleviate the problems of vanishing gradients and unstable training. Finally, the final stage of MSD-Net includes a convolutional network layer and a long shortcut global residual learning module to reconstruct fog-free images. These modules are responsible for further processing the features extracted, and then fused multiple times to reconstruct the image and restore clarity.

### 3.4. Loss Function

The L1 loss function, also known as the absolute value loss function, is one of the most commonly used loss functions. In remote sensing image dehazing tasks, the L1 loss function is used to measure the degree of difference between the generated dehazing image and the true clear image. Its function is to perform the following: (1) Provide pixel level consistency: The L1 loss function promotes the generated dehazing image to be consistent with the real image at the pixel level. It penalizes the absolute difference between each pixel in the predicted image and the corresponding pixel in the real image, thereby promoting the generated image to be closer to the real image; (2) Preserve detailed information: Compared to other loss functions, such as L2 loss function, L1 loss function is more sensitive to outliers. This means that the L1 loss function is more capable of preserving detailed information and avoiding excessive smoothing of the generated image in defogging tasks. Therefore, using the L1 loss function can better restore the detailed information in remote sensing images. Advantages compared to other loss functions: (1) Better noise resistance: L1 loss function is more robust to noise compared to L2 loss function. In remote sensing images, there may be interference from sensor noise or atmospheric scattering, and the L1 loss function can better handle these noises and reduce their impact on the dehazing effect; (2) Promote faster convergence: The L1 loss function can promote faster convergence speed during the training process. Compared to the L2 loss function, the L1 loss function has a greater response to gradients during the optimization process, allowing the network to learn appropriate weights and parameters more quickly; (3) Preserve texture details: Due to the nature of the L1 loss function, it tends to produce sparsity. This means that the generated dehazing image is more likely to retain the texture details in the original image, avoiding blurring or smoothing.

It is worth noting that the L1 loss function also has some limitations, such as sensitivity to outliers and significant gradient changes. Although many dehazing algorithms use perceptual loss and structural similarity loss, we still prioritize using L1 loss for training in MSD-Net.

(1) $$\mathrm{loss}(h,g)=\sum_{i=1}^{n}||gi-MSD(hi)||$$

Here, *h* and *g* represent the input parameters; *gi* represents the real situation of the ground, and *hi* represents the input of foggy image.

## 4. Experiment

### 4.1. Data Setting

In the task of remote sensing image defogging, it is difficult to obtain the real-world foggy image and the corresponding non-foggy image. Therefore, most defogging tasks are data driven and based on atmospheric scattering models via scattering coefficient β and atmospheric light intensity a to synthesize foggy image dataset. Remote sensing image defogging uses the open-sourced dataset HRRSD, which is the dataset released by the University of the Chinese Academy of Sciences in 2019 and contains 21,761 images obtained from Google Earth and Baidu maps. There are 55,740 target instances in HRRSD, and each category is about 4K. The HRRSD contains 13 categories of targets. The 13 categories are as follows: aircraft, baseball field, basketball court, bridge, intersection, track and field, port, parking lot, ship, storage tank, T-junction, tennis court, and car. The highlight of the dataset is that the sample size of each category is relatively balanced, and each category has about 4000 samples.

The HRRSD dataset is preprocessed and simulates the process of haze formation using an atmospheric scattering model. The formula for this model is as follows:

(2) I (x)=J (x) t (x)+A (1-t (x))

I (x) represents the synthesized foggy image mapping; J (x) represents the mapping of non-foggy targets; t (x) represents the medium transmission map dependent on unknown depth information, and A represents the global atmospheric light value. Haze remote sensing images are simulated by setting the transmittance t (x) and atmospheric light value A. 

In order to verify the generality of our model, we also selected the rice dataset in the field of remote sensing, which contains 500 foggy remote sensing satellite images and 500 non-foggy satellite images. We selected 450 pairs of images as the training dataset and 50 pairs of images as the validation dataset to evaluate the generality of our network model.

### 4.2. Training Setup

We train our model in the RGB channel and enhance the training dataset by randomly rotating the images to 90, 180, 270 degrees, and flipping them horizontally. Two blurry image blocks with a size of 256 × 256 are extracted as inputs for the MSD network. The entire network is trained in 5 × 105 steps. We use the Adam optimizer, where β1 and β2 take the default values of 0.9 and 0.999. The initial learning rate is set to 1 × 10^−4^, and we use cosine annealing strategy to adjust the learning rate from the initial value to zero by following the cosine function, assuming the total number of batches is T, η. If it is the initial rate of return, then at batch t, the learning rate ηt is calculated as follows:

(3) $$\eta t=\frac{1}{2}(1+\cos\left(\frac{t\pi }{T}\right))$$

Our model was implemented in Python on the Tesla T4 GPU. To assess the performance, we utilized peak signal-to-noise ratio (PSNR) and structural similarity index (SSIM) as our quantitative evaluation metrics. Higher PSNR and SSIM values indicate superior restored image quality. Additionally, a lower LPIPS value signifies greater similarity between two images. The PSNR formula is as follows:

(4) $$PSNR=10\times \log\left(\frac{Max_{I}^{2}}{MSE}\right)$$

Among these metrics, $Max_{I}^{2}$ denotes the maximum pixel intensity in the image, while MSE stands for the mean squared error between two images. 

SSIM takes into account three essential aspects of an image: luminance, contrast, and structure. The formula for SSIM is as follows:

(5) $$SSIM\left(x,y\right)=\frac{\left(2u_{x}u_{y}+C_{1})(2\sigma_{xy}+C_{2}\right)}{\left(u_{x}^{2}+u_{y}^{2}+C_{1})(\sigma_{x}^{2}+\sigma_{Y}^{2}+C_{2}\right)}$$

In this context, u represents the mean; $\sigma_{xy}$ denotes the covariance; $\sigma^{2}$ signifies the variance, and *C*_1_ and *C*_2_ are constants that remain unchanged.

### 4.3. Experimental Details

SCANet and GCANet are both deep learning networks based on attention mechanisms, and they have their own characteristics and application scenarios in feature extraction. SCANet is mainly used for 3D object detection, which enhances feature representations at different scales through spatial channel attention mechanism. It uses pyramid pooling and global average pooling to generate attention maps of space and channels, highlighting important features and suppressing unimportant information. In addition, SCANet also adopts an extended spatial upsampling model to restore spatial information and improve the accuracy of the 3D region proposal network. GCANet focuses on image dehazing and rain removal tasks. It encodes global information through global correlation operations so that the features of each location can contain global context, thereby more accurately estimating the dehazing effect of the image. GCANet also uses smooth dilated convolutions to reduce artifacts and employs gated fusion subnetworks to fuse multi-scale features, further improving dehazing performance. Their common core idea is to use attention mechanisms to enhance the model’s ability to capture key features, thereby improving performance in their respective fields. SCANet improves the accuracy of 3D detection through spatial and channel attention, while GCANet enhances the dehazing effect of images through global correlation and multi-scale feature fusion. Both models demonstrate the powerful potential of attention mechanisms in feature extraction.

Table 1 presents the quantitative evaluation results for the HRRSD and RICE datasets, including PSNR and SSIM metrics to measure the dehazing performance under different haze density conditions. On both datasets, our model has shown top-tier dehazing capabilities. Although our approach exhibits a slightly reduced dehazing effect in dense fog compared to thin fog, it still performs admirably in dealing with more severe image degradation, such as removing heavy haze from remote sensing images. Compared to other methods, SCANet has a relatively weaker defogging performance, while GCANet [17] has achieved a better defogging effect. Nevertheless, these methods still fall short in terms of the fineness of the dehazing results when compared to our model.

To further highlight the competitiveness of our model, we have chosen the MAXIM [37] model as a reference for comparison. MAXIM has already made significant achievements in the field of dehazing and serves as an advanced benchmark within the industry. Its performance is crucial for assessing the strengths and weaknesses of our model. By comparing with the MAXIM model, the unique advantages and potential of our model in handling complex foggy scenes become more pronounced. In summary, these results fully demonstrate that our model not only remains competitive in dehazing performance but also surpasses existing advanced methods in certain scenarios.

### 4.4. Qualitative Analysis

**Figure 3:**
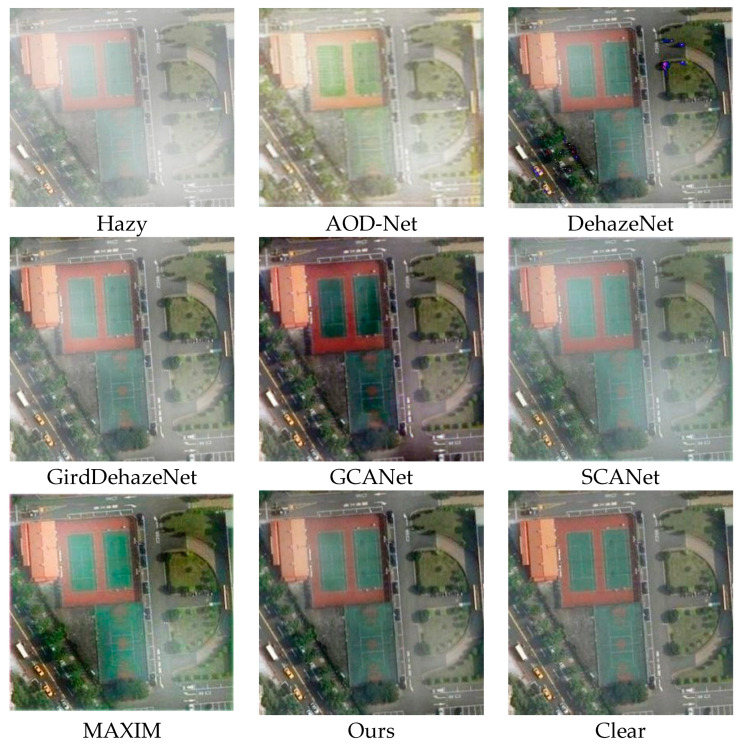
Visualization table of shallow concentration haze on HRRSD dataset.

In this section, we present a qualitative comparison of our model with five other advanced dehazing methods using the HRRSD and RICE remote sensing haze image datasets. Figure 3 illustrates the qualitative results of each method on the haze test. AOD-Net exhibits significant residual haze and color distortion in dehazing images, resulting in poor overall appearance. DehazeNet dehazing effect was not significant, with over half of the area still experiencing haze. SCANe is generally similar to the actual situation on the ground, but it can be observed that the haze has decreased, but it has not been completely removed. GridDehazeNet managed to achieve a certain level of dehazing, yet when compared to the ground truth images, a slight amount of haze remains in the overall restoration. GCANet demonstrated relatively good defogging capabilities. However, the images restored using GCANet appeared significantly darker than the ground truth, for instance, the runway in the playground appeared as dark red. Our method produced dehazing images that closely resemble the ground truth, achieving superior detail restoration.

**Figure 4:**
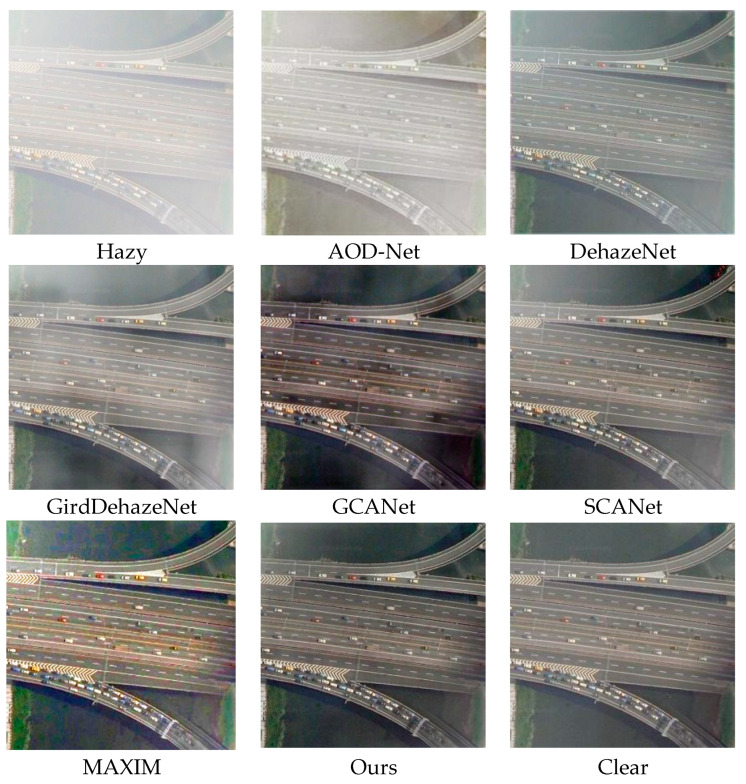
Visualization table of equal concentration haze in HRRSD dataset.

Figure 4 illustrates the comparative outcomes of various dehazing techniques when applied to images with moderate haze levels. Such levels of atmospheric turbidity tend to obscure critical details in satellite imagery. In this scenario, AOD-Net’s effectiveness in removing haze is subpar, leaving considerable fog in the enhanced image and resulting in noticeable discrepancies compared to the actual, undistorted scene. DehazeNet has a certain degree of haze removal effect, but there are still significant haze residues in important local information areas. SCANet has a relatively significant effect on removing haze, but there is a slight color distortion in certain areas. Compared to thin fog, GirdDehazeNet has a poorer dehazing effect in moderate concentration haze and can only remove a small amount of haze in some areas. Although GCANet has achieved large-scale defogging, a small amount of residual haze is more noticeable. In contrast, our method achieved excellent defogging performance under moderate haze conditions, exhibiting better color fidelity.

**Figure 5:**
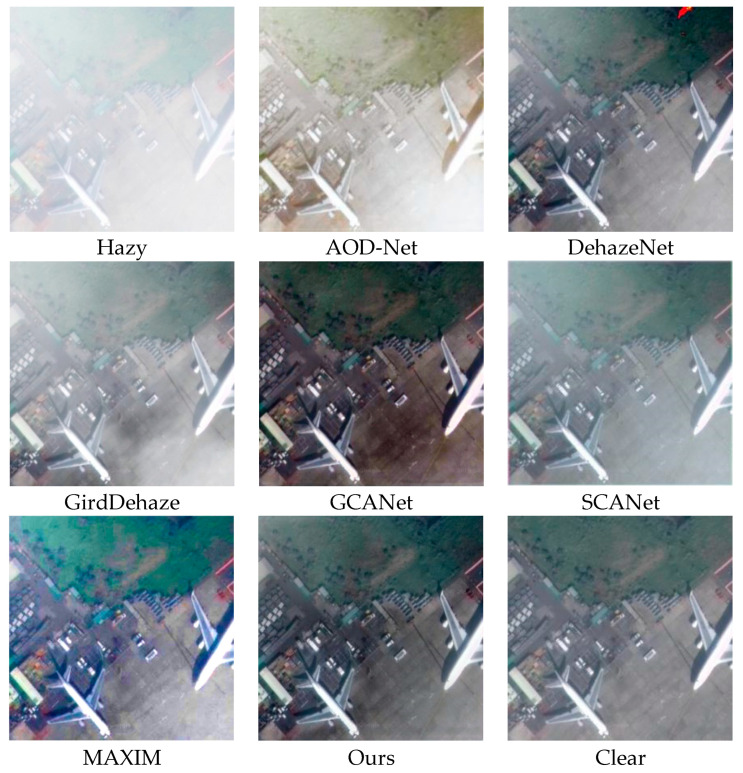
HRRSD dataset dense haze visualization table.

Figure 5 presents the qualitative analysis of different dehazing approaches when dealing with images affected by dense haze. The dataset predominantly features remote sensing photographs that have been significantly obscured by thick haze, leading to a substantial loss of texture and detail. The hazy images show that objects such as airplanes are nearly indistinguishable due to the haze. Under these dense haze conditions, AOD Net’s performance is unsatisfactory. While it manages to eliminate a thin layer of haze, the resulting image has a yellowish hue. Moreover, when compared to the ground truth, the restored image lacks accurate color representation, with the original color details being nearly unrecognizable. Although DehazeNet achieves a certain degree of dehazing effect, there is color distortion in some areas, which affects the overall appearance of the image. The dehazing effect of SCANet is average, and it can achieve a state of output from dense fog to thin fog. The images restored using GirdDehazeNet have a large amount of haze residue, especially in the grassland area, where the haze is more pronounced. GCANet can achieve complete dehazing, but the color is darker compared to the real ground conditions, and it appears black in the airport airspace area, which affects the overall appearance of remote sensing images. Our model still maintains good dehazing performance in dense haze conditions, which is close to the actual ground conditions.

**Figure 6:**
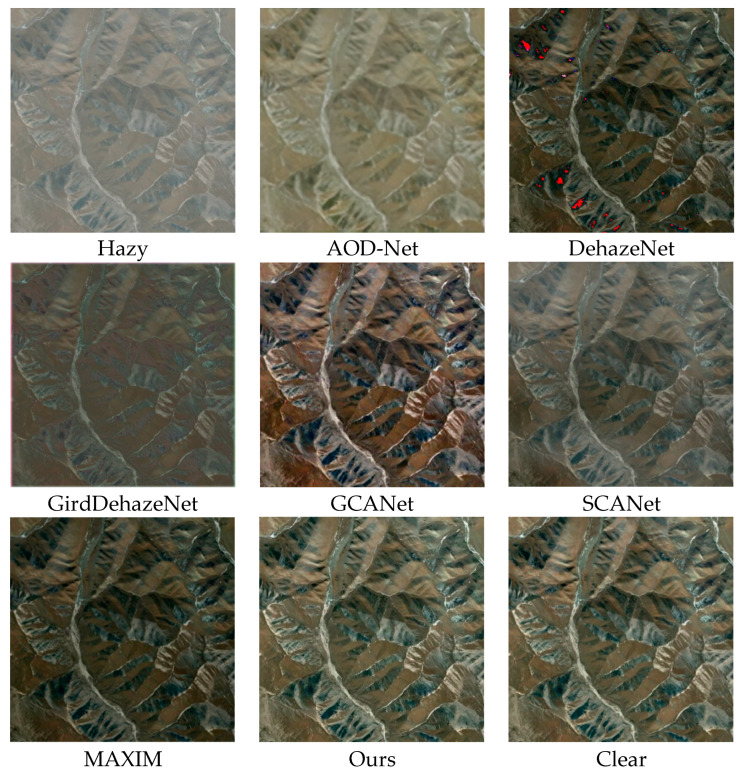
RICE dataset visualization table.

Figure 6 show the qualitative results of each method on the RICE test set, which is composed of dense and uniform remote sensing images obscured by haze. The effect of AOD-Net is poor. Although it has achieved a certain degree of defogging effect, the overall color of the mountains has changed, which is significantly different from the real situation on the ground. The image restored by DehazeNet has obvious dark tones, and there is local color distortion in the mountain area, which affects the overall appearance of the image. The dehazing effect achieved by SCANet is relatively poor, only achieving a small portion of dehazing. From an overall perspective, it only weakens the density of some haze. The image generated by GridDehaze has a darker central part and an overall white tone, with poor dehazing effect, only deepening the color of the mountain. The images restored by GCANet exhibit significant excessive dehazing and loss of original texture details. Compared with ground truth images, our model solves the challenge of texture restoration, enhances image details, and improves the color saturation and quality of remote sensing images.

**Figure 7:**
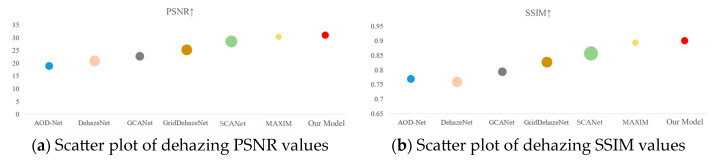
RICE Dataset Visualization Table.

To create a scatter plot for the evaluation metrics of the synthetic datasets, HRRSD_Dehaze and RICE were used, visually demonstrating the performance of various algorithms. The diameter of each scatter point represents the range from the maximum to the minimum value of each algorithm, as shown in Figure 7.

### 4.5. Ablation Analysis

To validate the effectiveness of our proposed method, we conducted ablation experiments on the HRRSD dataset to analyze the performance of our designed self-adaptive attention mechanism and multi-scale feature extraction using dilated convolution. The quantitative evaluation results are shown in Table 2. 

Initially, we established a foundational network devoid of self-adaptive attention and dilated convolution, denoted as “Base” in Table 2. Subsequently, we incrementally integrated the self-adaptive attention mechanism and dilated convolution into this foundational network, resulting in the configurations “Base + SA”, “Base + DC”, and “Base + SA + DC” as listed in Table 2. This process aimed to assess the individual contributions of these components to the network’s dehazing capabilities.

**Figure 8:**
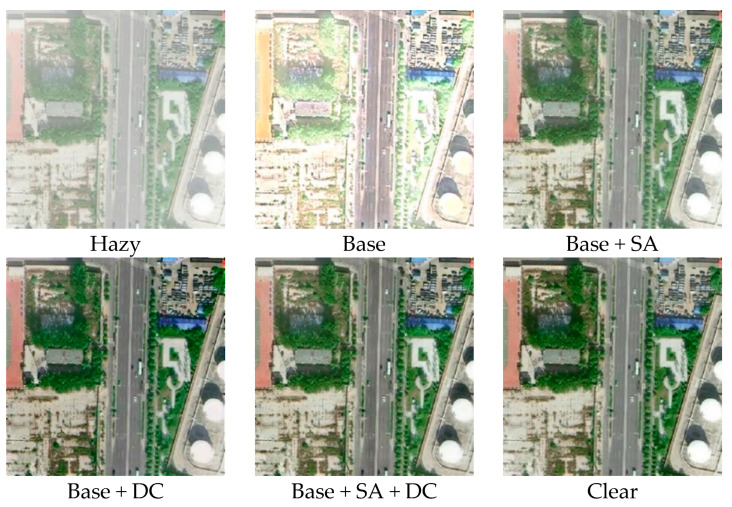
Visualization of ablation experiments on the HRRSD dataset.

Figure 8 visually contrasts the quantitative outcomes of our ablation study across various network modules, providing a clear demonstration of each module’s efficacy within our network. Drawing from the ablation study results on the HRRSD dataset as presented in Table 2 and Figure 6, we can deduce the following: The proposed method yields the poorest results in the absence of both self-adaptive attention and dilated convolution, leading to significant image distortion and subpar quality. Incorporating the self-adaptive attention mechanism into the Base network enhances PSNR and SSIM values, thereby improving image quality. Studies indicate that self-adaptive attention mechanisms adeptly concentrate on crucial image elements. However, they fall short in replicating the fine details and textures present in the actual ground truth. The addition of dilated convolution to the Base network introduces the multi-scale feature extraction that we designed. This modification results in a notable increase in both PSNR (by 1.46) and SSIM (by 0.115) compared to the Base network. The image quality is markedly enhanced, confirming that our dilated convolution-based multi-scale feature extraction effectively bolsters the network’s dehazing performance and restores the fine details of hazy remote sensing images. When both the self-adaptive attention mechanism and dilated convolution are integrated into the Base network, PSNR and SSIM values show further improvement over the Base + SA and Base + DC networks. This outcome validates the effectiveness of our multi-scale adaptive network design, which leverages dilated convolution to enhance image details and quality, ensuring a close resemblance to ground truth images.

## 5. Conclusions

In this article, we propose a multi-scale self-adaptive attention network to solve the problem of dehazing in remote sensing images. Our model includes a multi-scale adaptive feature extraction module consisting of dilated convolutions with different expansion rates and self-adaptive attention mechanisms, expanding convolutions with different expansion rates to obtain multi-scale information and preserve detailed features. The self-adaptive attention mechanism adaptively processes the uneven distribution of haze and important feature information in remote sensing images and outputs useful features to the network backbone. The multi-scale adaptive feature extraction module can focus and enhance the main information during the defogging process, further improving the defogging quality of remote sensing images. The experimental results show that this method achieves state-of-the-art dehazing effects on HRRSD and RICE benchmark remote sensing fuzzy datasets and significantly restores the detailed information of the image. In future work, we plan to design a new remote sensing image dehazing algorithm and construct a more realistic public large-scale remote sensing image dehazing dataset to promote research in the field of remote sensing image dehazing.

## Figures and Tables

**Table 1:** Quantitative comparison of dehazing results on HRRSD and RICE datasets.

Method	HRRSD				RICE			
	PSNR	SSIM	LOE	MAE	PSNR	SSIM	LOE	MAE
AOD-Net	21.46	0.808	0.076	0.375	17.05	0.731	0.085	0.362
DehazeNet	22.31	0.771	0.064	0.324	19.12	0.763	0.063	0.356
SCANet	24.67	0.813	0.065	0.321	22.81	0.796	0.074	0.305
GirdDehazeNet	26.12	0.864	0.054	0.347	25.75	0.870	0.065	0.285
GCANet	27.91	0.866	0.045	0.313	27.07	0.863	0.046	0.263
MAXIM	30.50	0.950	0.043	0.235	32.75	0.980	0.043	0.222
Ours	32.21	0.975	0.032	0.225	34.26	0.988	0.036	0.196

**Table 2:** Quantitative comparison of dehazing results on HRRSD and RICE datasets.

Method	HRRSD	
	PSNR	SSIM
Base	18.16	0.846
Base + SA	28.49	0.970
Base + DC	34.14	0.987
Base + SA + DC	36.85	0.998

## Data Availability

The data presented in this study are available on request from the corresponding author after obtaining permission from an authorized person.
